# Long non-coding RNA and microRNA-675/let-7a mediates the protective effect of melatonin against early brain injury after subarachnoid hemorrhage via targeting TP53 and neural growth factor

**DOI:** 10.1038/s41419-017-0155-8

**Published:** 2018-01-24

**Authors:** Song Yang, Wanzhong Tang, Yuchao He, Linbao Wen, Bin Sun, Shengli Li

**Affiliations:** 1 Department of Neurosurgery, Suqian First Hospital, Suqian, Jiangsu China; 2 Department of Neurosurgery, The Affiliated Hospital of Medical College Qingdao University, Qingdao, Shandong China; 3 Department of Neurosurgery, The Second Affiliated Hospital of Guiyang College of Traditional Chinese Medicine, Guiyang, Guizhou China; 4 Department of Neurosurgery, Qilu Hospital of Shandong University (Qingdao), Qingdao, Shandong China; 5 Department of Neurosurgery, Municipal Hospital, Qingdao, Shandong China

## Abstract

The objective of this study was to identify the protective effect of melatonin (MT) against early brain injury (EBI) following subarachnoid hemorrhage (SAH) and explore the underlying molecular mechanism. Real-time polymerase chain reaction (PCR) and luciferase assay were utilized to detect the effect of MT on H19 expression level, computation analysis and luciferase assay were conducted to the underlying mechanism of let-7a and miR-675. Real-time PCR, western blot analysis, immunohistochemistry, 3-(4,5-dimethylthiazol-2-yl)-2,5-diphenyltetrazolium bromide (MTT) assay, and flow cytometry analysis were performed to detect the effect of MT on H19, miR-675, let-7a, TP53, neural growth factor (NGF) levels, cell viability, and apoptosis status. Melatonin increased H19 expression level by enhancing H19 transcriptional efficiency in a concentration-dependent manner. MiR-675 and let-7a directly targeted P53 and NGF, respectively, and miR-675 reduced luciferase activity of wild-type but not mutant TP53 3′UTR. Meanwhile, let-7a suppressed luciferase activity of wild-type but not mutant NGF 3′UTR. H_2_O_2_ increased number of SA-b-gal, and while MT administration repressed the premature senescence. H_2_O_2_ obviously upregulated expressions of H19, miR-675, and NGF, and downregulated let-7a and TP53 levels; however, MT treatment reduced expressions of H19, miR-675, and NGF, and improved let-7a and TP53 levels. Treating with MT attenuated the neurological deficits and reduced the brain swelling. MT treatment repressed apoptosis of neurons caused by SAH. Levels of H19, miR-675, and NGF were much higher in the SAH +  MT group, while there were even higher levels of H19, miR-675, and NGF in the SAH group than in the sham group; levels of let-7a and TP53 were much lower in the SAH + MT group, while they were even lower in the SAH group than in the sham group. Our study revealed that treatment with MT protected against EBI after SAH by modulating the signaling pathways of H19-miR-675-P53-apoptosis and H19-let-7a-NGF-apoptosis.

## Introduction

Many medical conditions of central nervous system are potentially life threatening, and subarachnoid hemorrhage (SAH) is one of them. The World Health Organization (WHO) reported an incidence of 22.5 cases of SAH in 100,000 individuals each year^1^. Due to the poor outcomes of SAH, extensive research has been conducted to better understand the disease. One highly influential factor identified previously is early brain injury (EBI)^2^. Individuals presented with EBI following an event of SAH typically experience abnormally enhanced inflammatory response, oxidative stress, cerebral vasospasm, and cell death^3^.

One key factor that influences the development of EBI following SAH is apoptosis, or cell death, and neural growth factor (NGF) and TP53 are believed to be two of the most important regulators of cell apoptosis^4^. If NGF is knocked out, the neuron will be destined to undergo the apoptosis^5^. Neural growth factor is believed to suppress apoptosis of neurons by activating phosphatidylinositol 3-kinase (PI3K)/Akt signaling. This subsequently promoted the expression of pro-apoptotic Bcl-2 family members^6^. TP53 is the overall most important factor in initiating the apoptosis process in response to ischemia, hypoxia, or severely damaged DNA, and it has been well studied as a tumor suppressor^7^.

Long non-coding RNAs (lncRNAs) is a class of non-coding RNA that is composed of more than 200 nucleotides (nt) that regulate the expression of gene(s) at transcriptional and post-transcriptional levels^8^. Long non-coding RNAs do not have any particular open reading frame or coding sequence^9^. Meanwhile, microRNAs (miRNAs) is a class of single-stranded, small non-coding RNAs consisting of between 21 and 24 nt, and they are involved in the control of multiple cellular activities such as proliferation, differentiation, and apoptosis by regulating the expression of genes at the post-transcriptional or translational level^10^. MicroRNA-675 (miR-675) has been reported to be a derivative of H19^11^. MiR-675 has been reported to suppress placental growth and promote differentiation or regeneration of skeletal muscle cells^11,12^. Meanwhile, let-7a has been known to be involved in regulating the apoptosis of the cells^13^.

Some studies have indicated that MT may be useful in ameliorating EBI, and thereby could have a protective role in the management of SAH^14^. In addition, MT has also been found to be able to prevent vasospasm, decrease mortality, and reduce apoptosis of neurons^15,16^. It has also been indicated that it can alleviate focal cerebellum injury^17,18^. Melatonin has been reported to protect brain against post-SAH injury^19^. It has been also found that administration of MT could alter the expression of lncRNA and H19, and H19 has been shown to host miR-675, and miR-675 has been found to be a negative regulator of P53^20,21^. In another study, H19 was found to be a competing non-coding RNA for let-7a, and let-7a was found to be a virtual regulator of NGF by using an online miRNA database (www.mirdb.org)^22^. In this study, we established animal model of SAH, which was treated with MT, and the possible involvement of H19-miR-675-P53-apoptosis and H19-let-7a-NGF-apoptosis signaling pathway was investigated in those animals.

## Materials and methods

### Animals

Healthy adult male C57BL/6J mice were used in this study. The mice were purchased from the experimental animal center of our institute and weighed between 22 and 25 g. All the experiments were carried out under the guidance of the Guide for the Care and Use of Laboratory Animals which was published by NIH and was approved by the Ethics Committee of our institute.

### SAH model

The endovascular perforation method was used to establish animal model of SAH. Pentobarbital sodium (50  mg/kg) was injected to perform anesthetization. A heat blanket was used to maintain the rectal temperature at 37 ± 0.5 °C. The internal carotid artery, external carotid artery, and left common carotid artery were exposed after a midline incision at the neck. Ligation and cut at the left external carotid was finished to leave a 3-mm stump. Insertion of a nylon suture into the left internal carotid artery was accompanied to perforate the artery at the bifurcation of the anterior and middle cerebral artery. The identical procedures were performed on Sham-operated mice except the artery perforation.

### Experimental protocol

The mice were assigned into the groups listed below: (1) SAH group (*n* = 12), (2) sham group (*n* = 12), (3) SAH + MT group (*n* = 12). Melatonin was dissolved in 1% ethanol in 1 ml saline, then Mel was intraperitoneally injected at a dose of 150 mg/kg 2 and 12 h after SAH.

### Neurological score

Neurological deficits were assessed with an 18-point system from six tests at 24 h after SAH; the six tests are: forelimbs outstretching (0–3), spontaneous activity (0–3), climbing (1–3), symmetry in the movements of all limbs (0–3), response to vibrissae touch (1–3), and body proprioception (1–3). A higher score indicates an elevated function.

### Brain water content

The standard wet–dry method was used to measure the brain water content. Briefly, mice were killed, and mice brains were immediately isolated and separated into the right and left cerebellum, cerebral hemispheres and brain stem; these parts were weighed respectively to obtain wet weight. Then, brain sections were dried for 24 h at 105 °C and weighed to obtain the dry weight. Finally, the brain water content was inferred as follows: (wet weight -dry weight)/wet weight × 100.

### RNA isolation and real-time PCR

Total RNA extraction was performed on ancerous/non-cancerous specimens or cell lines with TRIzol® reagent (Invitrogen) following instructions indicated by the supplier. Reverse transcription of the total RNA into TP53 or NGF cDNA was accompanied with the reverse transcription kit with random primers (Invitrogen). Primers specific to H19, TUG1, SNHG14, MEG3, let-7a, miR-675, TP53 mRNA, NGF mRNA were designed and used to evaluate the expression level of these genes by real-time qPCR with SYBR® Green real-time PCR kit (Toyobo, Co., Ltd., Osaka, Japan). Each reaction contains 10 μl of PCR mix, 5 pmol of forward and reverse primers, 1 μl cDNA template and proper volume of water to achieve a 20 μl total reaction volume. Each reaction was replicated for at least three times. The reaction was performed on a Roche 480 system under conditions listed as follows: initial denaturation at 94 ˚C for 3 min; 45 cycles of denaturation at 94 ˚C for 20 s, annealing at 60 ˚C for 60 s, and elongation at 72 ˚C for 45 s; and final elongation at 72 ˚C for 3 min. The 2^−ΔΔCT^ method was used to calculate the relative expression of genes and miRNA, including H19, TUG1, SNHG14, MEG3, let-7a, miR-675, TP53 mRNA, NGF mRNA.

### Cell culture and transfection

U87 and U251 cells were cultured in DMEM high glucose medium containing 10% Fetal Bovine Serum(FBS) and maintained at 37 °C in a humidified incubator with 5 % CO_2_. Cells were seeded into six-well dishes and treated with MT at a final concentration of 100 nm, 1 μm, and 10 μm, respectively. When the cells were grown to 70–80% confluence, lipofectamine^2000^ transfection reagent (Invitrogen, California, USA) was used to transfect the U87 and U251 cells with let-7a or miR-675 based on the standard protocol by the supplier. All experiments were carried out three times.

### Cell proliferation assay

The cells were seeded in a 12-well dish and incubated overnight, and the cells were incubated with or without MT for 48 h. The proliferation rates of the cells were measured with MTT assay (Sigma-Aldrich, Inc., St. Louis, MO, USA) according to the manufacturer’s instructions. Briefly, 50 mg MTT was used to treat cells in each well, dimethyl sulfoxide (DMSO) was used to dissolve the formazan, then a plate reader was used to measure the absorbance at 590 nm. The experiments were repeatedly carried out for three times.

### Vector construction and mutagenesis, and luciferase assay

3′UTRs of TP53 and NGF were cloned using primers specific to these fragments. Site-directed mutagenesis of miR-675 binding sites in the 3′UTR of TP53 and let-7a binding sites in the 3′UTR of NGF was performed by using site-Directed Mutagenesis Kit (SBS Genetech). The wild-type and mutant 3′UTRs of TP53 or NGF was cloned into pGL3 at the downstream of the firefly luciferase reporter gene. U87 and U251 cells were seeded in a 24-well plate, co-transfection of miR-675, the luciferase expressing plasmid pRL-TK and TP53 reporter plasmids containing wild-type or mutant 3′UTR were performed. Similarly, co-transfection of let-7a and NGF reporter plasmids was carried out in cells. After 48 h incubation, the cells were harvested and the assay was performed using D-20/20 luminometer (Turner Biosystems).

### Vector construction and mutagenesis and luciferase assay

Full fragment of the promoters of H19, TUG1, SNHG14 or MEG3 was amplified using PCR. The products were inserted into pGL3 at the upstream of the firefly luciferase reporter gene. U87 and U251 cells were seeded in a 24-well plate, and transfected with constructs containing the promoters of H19, TUG1, SNHG14 or MEG3. U87 and U251 cells were subjected to treatment with MT at a final concentration of 100 nm, 1 μm, and 10 μm, respectively. Luciferase activity of H19, TUG1, SNHG14 or MEG3 was determined using D-20/20 luminometer (Turner Biosystems).

### Western blot analysis

To detect and compare the expression of TP53 and NGF protein levels, U87 and U251 cells were collected and lysed in lysis buffer (1 mm EDTA, 50 mm Tris pH 7.4, 250 mm NaCl, 0.1% NP40, 5 mm sodium fluoride (NaF), 0.5 mm NaVO_3_ pepstatin, benzamidine, aprotinin, phenylmethanesulfonylfluoride or phenylmethylsulfonyl fluoride (PMSF), leupeptin). The cell extracts were mixed with 0.01% bromophenol blue and 5% 2-Mercaptoethanol and boiled in a water bath. A total amount of 40 mg proteins were separated by PAGE:sodium dodecyl sulfate polyacrylamide gel electrophoresis (SDS)-PAGE and further transferred onto polyvinylidene difluoride (PVDF) membrane. phosphate buffered saline-tween (PBS-T)-dissolved 5% fat-free milk was used to block the membranes for 2 h in darkness, following which the primary antibodies against TP53 at a dilution of 1:2000 (Santa Cruz Biotechnology, CA, USA) or NGF at a dilution of 1:5000 (Santa Cruz Biotechnology, CA, USA) were incubated with the membranes at 4 ℃ for 12 h. Monoclonal anti-β-actin (Cell Signaling) was selected as the loading control. Visualization of the immune complexes was further detected with horseradish peroxidase-conjugated antiserums (Santa Cruz Biotechnology). The photographed with Chemi- Doc™ XRS System (BIO-RAD, USA) and immobilon western chemiluminescent HRP Substrate (Millipore, Billerica, MA, USA) were used to visualize the immune-complexes.

### Immunohistochemistry

The immunoreactivity of TP53 and NGF is determined by using immunohistochemistry. Briefly, the sections were firstly deparaffinized and rehydrated in ethanol of graded concentrations to water. Five-minute treatment of 3% H_2_O_2_ was conducted to block the endogenous peroxidase activity, followed by water rinse and PBS washing for 15 min. Sections were further placed in citrate buffer and heated for 30 min at 95 °C in microwave oven, then cooled at RT for 20 min and rinsed in PBS. Forty-minute incubation with 5% horse serum was performed to block the nonspecific protein binding. Primary antibodies for TP53 (diluted 1:500; Santa Cruz Biotechnology) and NGF (diluted 1:500; Santa Cruz Biotechnology) were diluted and incubated with the sections at RT for 1 h. Followed by PBS washing and incubation with HRP-conjugated IgG (1:1000 dilution; Santa Cruz Biotechnology) at RT for 1 h. Hematoxylin was used to perform counterstaining with 3'-diaminobenzidine (DAB) as chromogen.

### Terminal deoxynucleotidyl transferase dUTP nick end labeling (TUNEL)

The apoptosis status of the brain cortex was evaluated using TUNEL assay following the instructions provided by the manufacturer. The ratio was calculated as number of TUNEL-positive neurons /total neurons.

### Statistical analysis

All data analyses were performed with SPSS 21.0 software (IBM, Chicago, IL), and Chi square test, *t*-test, or one-way ANOVA were used to examine the difference of the study targets among the groups. *P < *0.05 was considered statically significant.

## Results

### Melatonin affected H19 level by influencing H19 promoter transcription efficiency

**Fig. 1:**
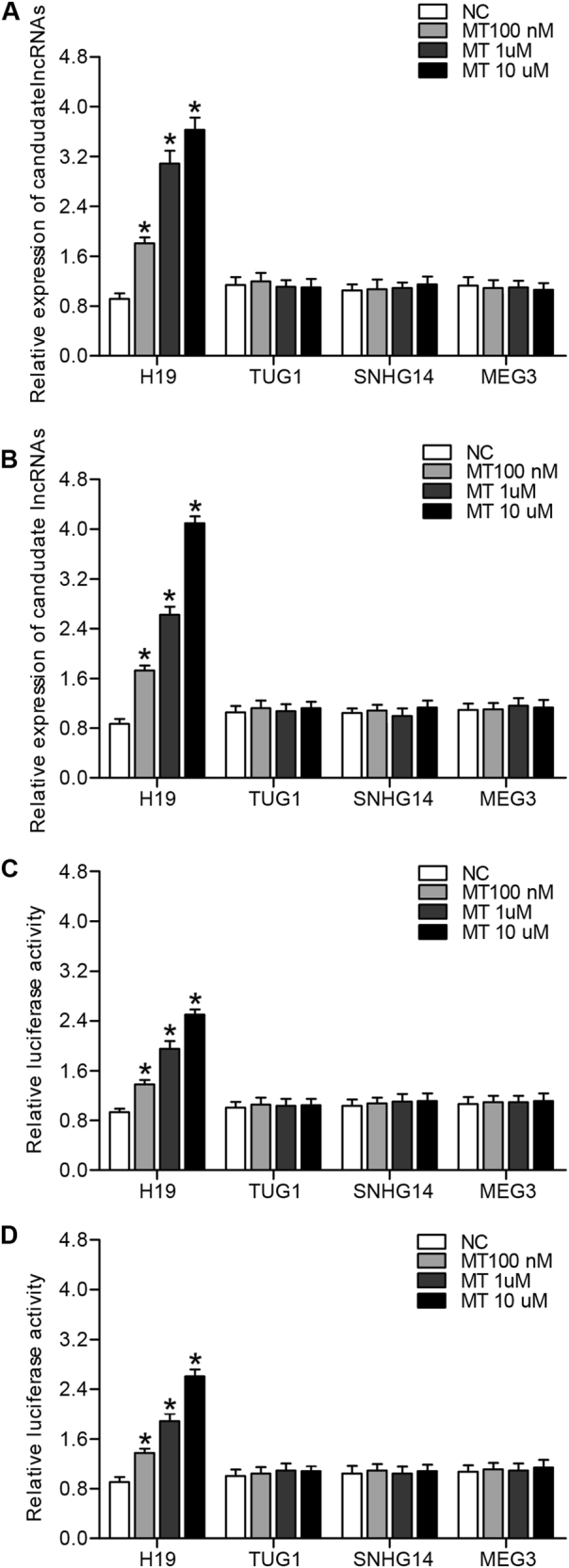
MT modulated H19 level by affecting H19 promoter transcription efficiency (*P < 0.05 compared with the control) **a** Only H19 among H19, TUG1, SNHG14, MEG3 was increased following MT treatment, and under a concentration manner in U87 cells. **b** Only H19 among H19, TUG1, SNHG14, MEG3 was dose-dependently increased following MT treatment in U215 cells. **c** Only luciferase activity of H19 among H19, TUG1, SNHG14, MEG3 was dose-dependently upregulated subsequent to treatment with MT in U87 cells. **d** Only luciferase activity of H19 among H19, TUG1, SNHG14, MEG3 was dose-dependently upregulated subsequent to treatment with MT in U251 cells

Real-time PCR and luciferase assay were utilized to explore the effect of MT on H19 transcriptional efficiency. Different doses of MT (100 nm, 1 μm, 10 μm) were utilized to treat U87 cells and U251 cells. Then real-time PCR was performed to examine and compare the expression level of candidate lncRNAs including H19, TUG1, SNHG14, MEG3 between MT treated and untreated groups. As shown in Fig. 1, MT-treated U87 cells (Fig. 1a) and U251 (Fig. 1b) cells showed a stepwise increase in H19 level as the concentration of MT increased, while TUG1, SNHG14, MEG3 levels in MT-treated U87 (Fig. 1a) and U251 (Fig. 1b) showed no obvious difference between the two groups. Furthermore, luciferase assay was conducted to confirm whether MT affected H19 level by influencing H19 transcriptional efficiency, and luciferase activity of H19 in MT-treated U87 cells (Fig. 1c) or U251 cells (Fig. 1d) was increased in a concentration-dependent manner in comparison with the control.

### MiR-675 or let-7a directly targeted P53 or NGF, respectively

**Fig. 2:**
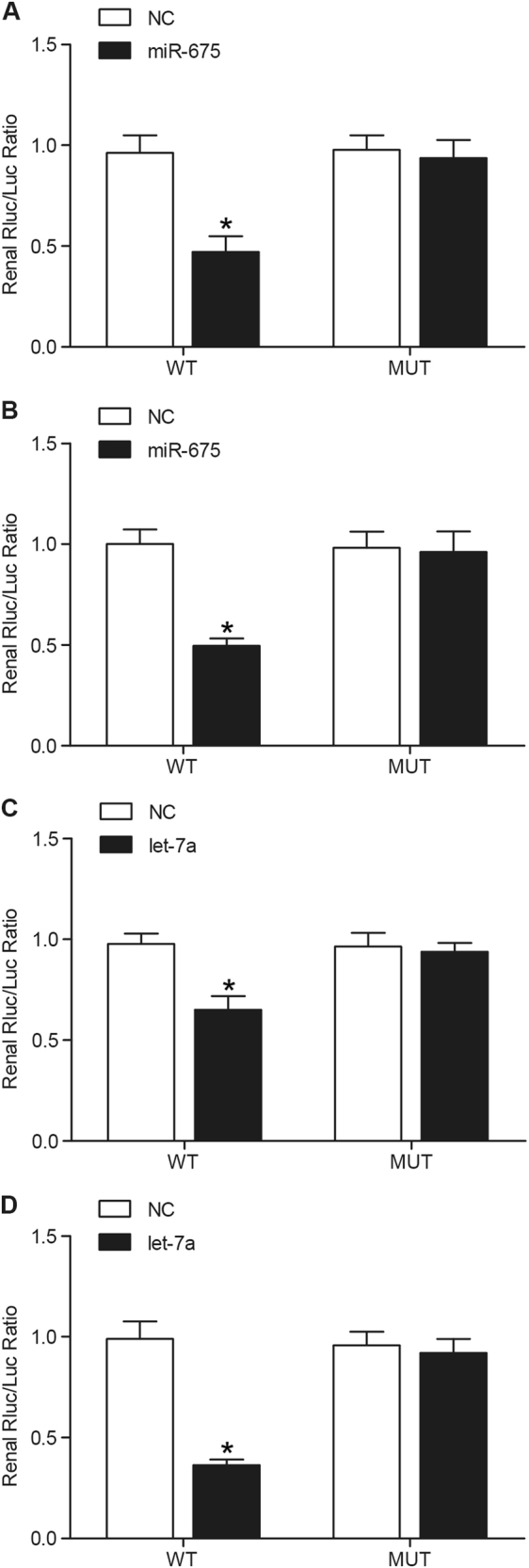
*P53* or *NGF* was candidate gene of miR-675 or let-7a, respectively (*P < 0.05 compared with the control) **a** miR-675 evidently inhibited luciferase activity of wild-type P53 3′UTR but not that of mutant P53 3′UTR in U87 cells. **b** miR-675 evidently inhibited luciferase activity of wild-type P53 3′UTR but not that of mutant P53 3′UTR in U251 cells. **c** Luciferase activity of wild-type but not mutant NGF 3′UTR in U87 cells transfected with let-7a mimic was much lower. **d** Luciferase activity of wild-type but not mutant NGF 3′UTR in U251 cells transfected with let-7a mimic was much lower

Both miR-675 and let-7a have been reported to be functionally associated with H19, and we further search the online miRNA database for the potential target of the miRNAs. We identified *P53* or *NGF* as the candidate target gene of miR-675 or let-7a respectively with the “seed sequence” in the 3′UTR. And luciferase assay results showed that miR-675 suppressed luciferase activity of wild-type but not that of mutant P53 3′UTR in either U87 cells (Fig. 2a) or U251 cells (Fig. 2b) compared to that in the control. Meanwhile, let-7a suppressed luciferase activity of wild-type but not that of mutant NGF 3′UTR in either U87 cells (Fig. 2c) or U251 cells (Fig. 2d) compared to that in the control, suggesting that *P53* was a direct target gene of miR-675, and *NGF* was a direct target gene of let-7a.

### MT affected cell apoptosis, H19, miR-675, let-7a, TP53, NGF levels

**Fig. 3:**
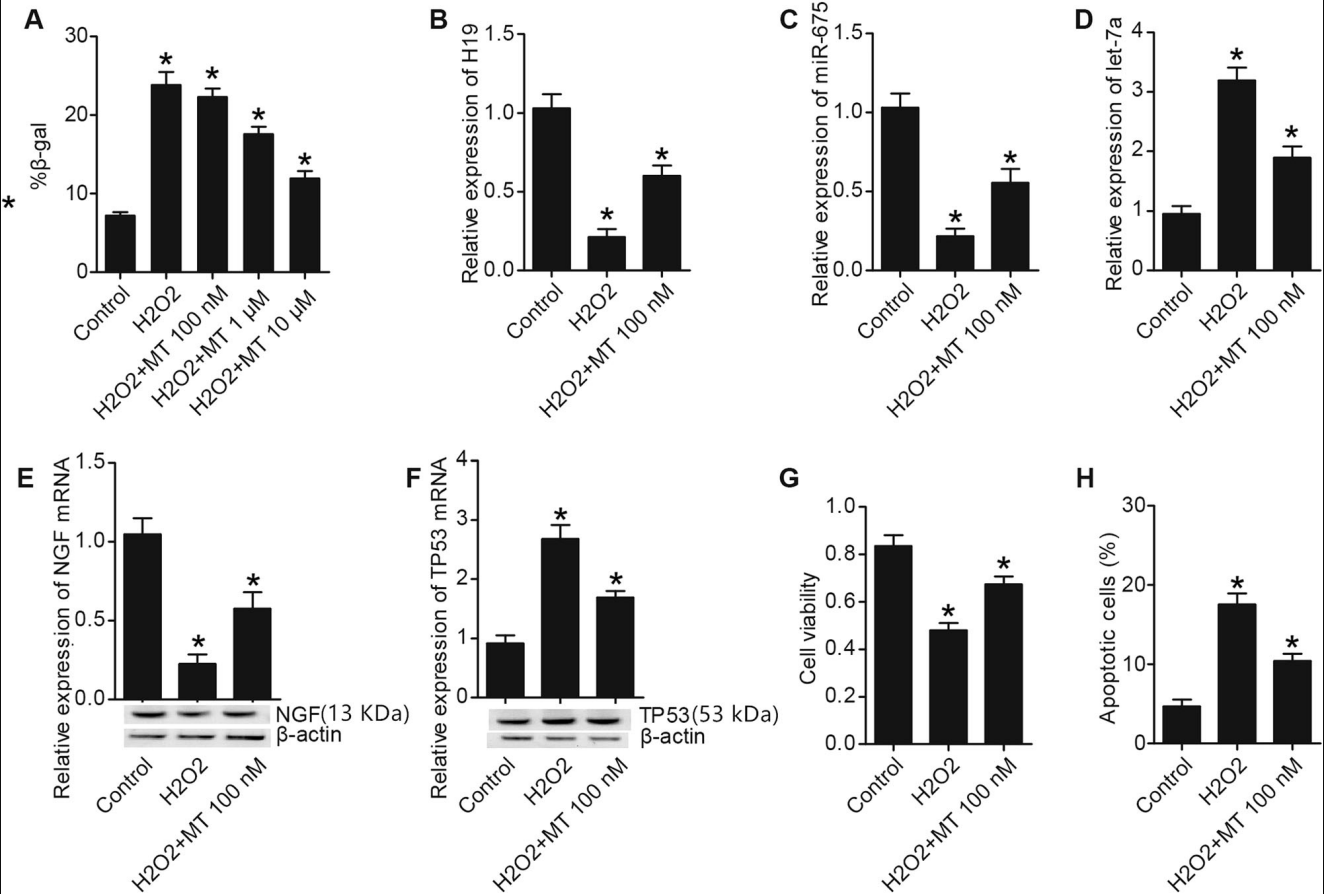
MT regulated cell apoptosis, H19, miR-675, let-7a, TP53, NGF levels in U87 cells (*P < 0.05 compared with the control) **a** H_2_O_2_ treatment increased number of SA-b-gal, which was restored by 1 and 10 μm of MT administration. **b** H19 level in H_2_O_2_ treatment group was much lower than H_2_O_2_ plus MT treatment group; both of them were much lower than control. **c** miR-675 level in H_2_O_2_ plus MT treatment group was much lower than control, and was even lower in H_2_O_2_ treatment group. **d** let-7a level in H_2_O_2_ treatment group was much higher than H_2_O_2_ plus MT treatment group; both of them were much higher than control. **e** NGF level in H_2_O_2_ plus MT treatment group was much lower than control, and was even lower in H_2_O_2_ treatment group. **f** TP53 level in H_2_O_2_ treatment group was much higher than H_2_O_2_ plus MT treatment group; both of them were much higher than control. **g** Treating with H_2_O_2_ or H_2_O_2_ plus MT inhibited proliferation of U87 cell, but the inhibition effect of H_2_O_2_ was much stronger than H_2_O_2_ plus MT. **h** Treating with H_2_O_2_ or H_2_O_2_ plus MT promoted apoptosis of U87 cell, but the promotion effect of H_2_O_2_ was much stronger than H_2_O_2_ plus MT

**Fig. 4:**
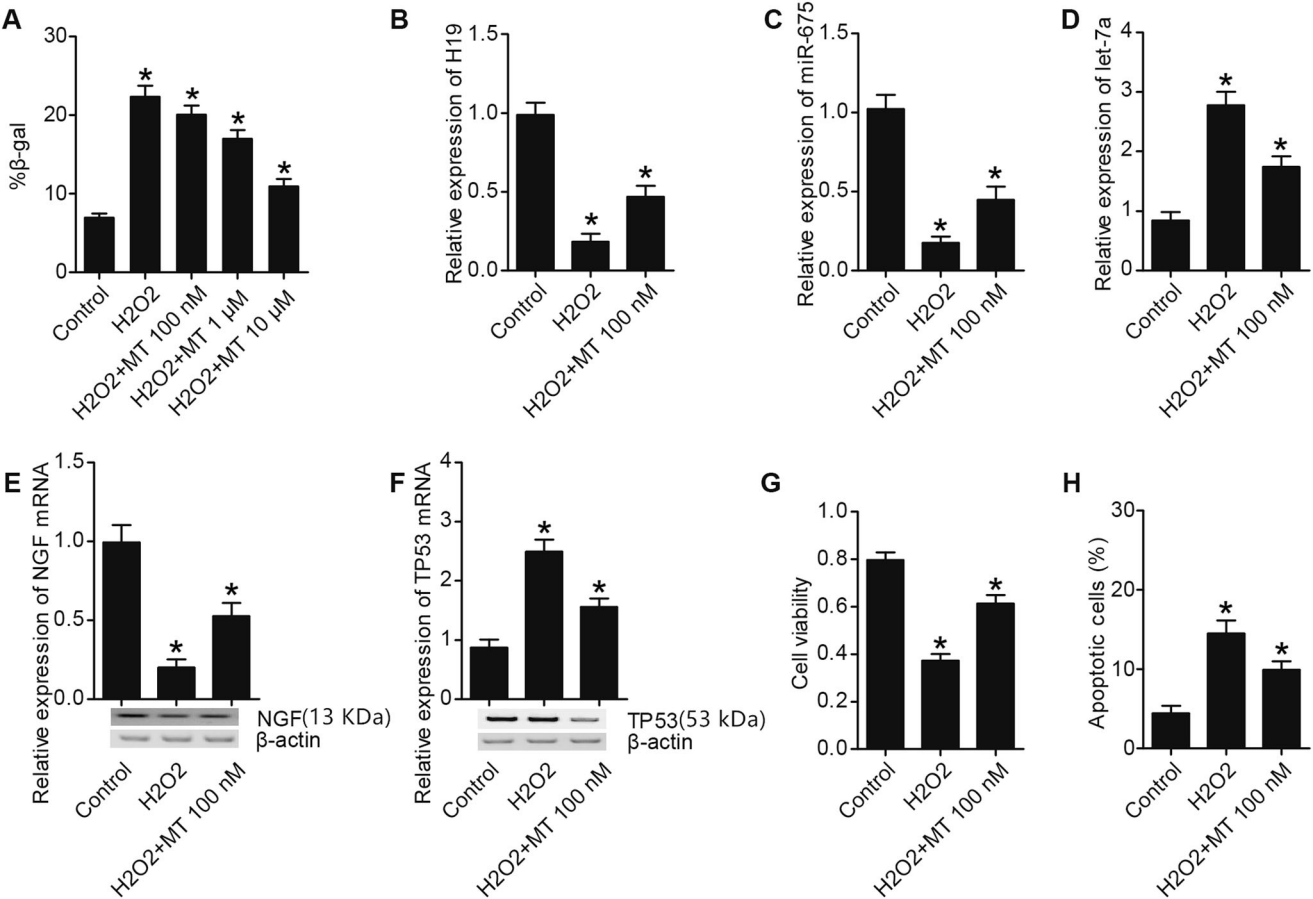
MT regulated cell apoptosis, H19, miR-675, let-7a, TP53, NGF levels in U251 cells (*P < 0.05 compared with the control) **a** H_2_O_2_ treatment increased number of SA-b-gal, which was restored by 1 and 10 μm of MT administration. **b** H19 level in H_2_O_2_ treatment group was much lower than H_2_O_2_ plus MT treatment group; both of them were much lower than control. **c** miR-675 level in H_2_O_2_ plus MT treatment group was much lower than control, and was even lower in H_2_O_2_ treatment group. **d** let-7a level in H_2_O_2_ treatment group was much higher than H_2_O_2_ plus MT treatment group; both of them were much higher than control. **e** NGF level in H_2_O_2_ plus MT treatment group was much lower than control, and was even lower in H_2_O_2_ treatment group. **f** TP53 level in H_2_O_2_ treatment group was much higher than H_2_O_2_ plus MT treatment group; both of them were much higher than control. **g** Treating with H_2_O_2_ or H_2_O_2_ plus MT inhibited proliferation of U251 cell, but the inhibition effect of H_2_O_2_ was much stronger than H_2_O_2_ plus MT. **h** Treating with H_2_O_2_ or H_2_O_2_ plus MT promoted apoptosis of U251 cell, but the promotion effect of H_2_O_2_ was much stronger than H_2_O_2_ plus MT

To verify the protective role of MT against apoptosis, sub-lethal concentration of H_2_O_2_ (<200 μm), or H_2_O_2_ along with various doses of MT was utilized to treat U87 and U251 cells. As shown in Figs. 3a and 4a, the number of SA-b-gal (senescence-associated b-galactosidase)-positive U87 (Fig. 3a) and U251 (Fig. 4a) cells treated with H_2_O_2_ were greater than control, while 1 μm and 10 μm of MT inhibited premature senescence of U87 cells (Fig. 3a) and U251 cells (Fig. 4a). Co-culture with H_2_O_2_ obviously upregulated expressions of H19 (Figs. 3b and 4b), miR-675 (Figs. 3c and 4c), NGF (Figs. 3e and 4e) in U87 cells (Fig. 3) and U251 cells (Fig. 4), as well as the viability of U87 cells (Fig. 3g) and U251 cells (Fig. 4g), which were partly restored by the administration of MT. By contrast, H_2_O_2_ remarkably inhibited expressions of let-7a (Figs. 3d and 4d), TP53 (Figs. 3f and 4f) in U87 cells (Fig. 3) and U215 cells (Fig. 4), as well as the apoptosis of U87 cells (Fig. 3h) and U251 cells (Fig. 4h), and the inhibition of H_2_O_2_ on let-7a, TP53, and cell apoptosis were partly abrogated by co-culture with MT.

### Melatonin provided the brain relief from edema and neurological deficits after SAH

**Fig. 5:**
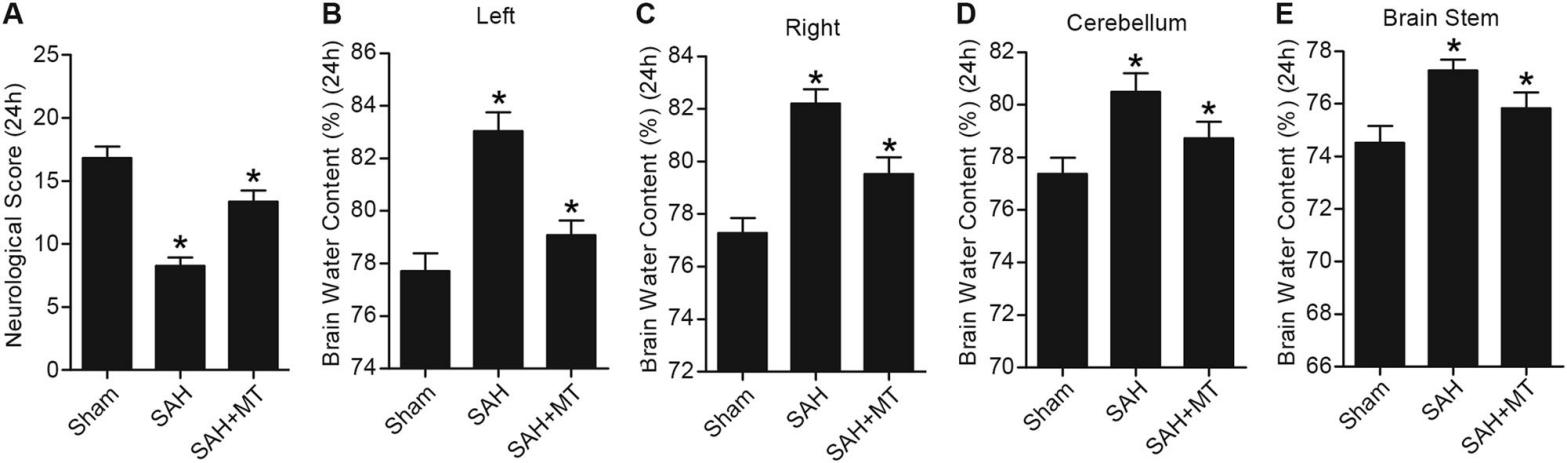
The effects of MT on water content of brain and neurological deficits (*P < 0.05 compared with the sham group) **a** SAH group displayed a lower level of water content in brain, and MT treatment improved neurological score. **b** Evident increase in brain swelling, including left, right, cerebellum, and brain stem (**b**−**e**) in the SAH group, which was improved by MT treatment

We further established the animal models of SAH, which were treated with administration of MT, and the brain water content and neurological scores were examined within 24 h following the SAH. We found that the mice in the SAH group displayed an evident decline in neurological score (Fig. 5a), as well as an obvious increase in water content of different anatomical brain structure including (Fig. 5b−e). We also noticed MT treatment improved the neurological deficits (Fig. 5a) and attenuated the brain swelling (Fig. 5b−e) 24 h following SAH event.

### MT inhibited apoptosis of neuronal in the brain cortex caused by SAH

**Fig. 6:**
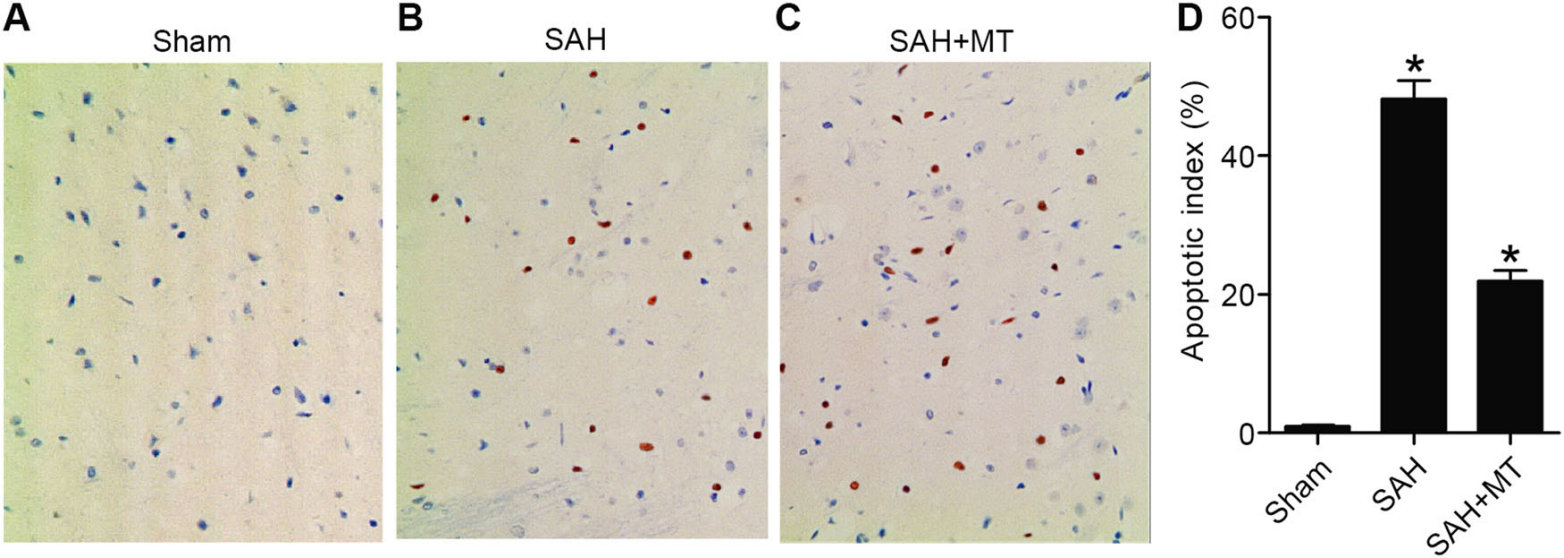
MT suppressed apoptosis of neuronal in the brain cortex induced by SAH **a** Apoptosis status in the sham group, **b** apoptosis status in the SAH group, **c** apoptosis status in the SAH group treated with MT, **d** summary and comparison between the sham group, SAH group, and SAH + MT group (*P < 0.05 compared with the sham group)

TUNEL assay was utilized to evaluate the apoptosis of the neurons. As shown in Fig. 6, evident increase in apoptotic index in the SAH group compared with the sham group. MT treatment remarkably repressed apoptosis of neurons in the brain 24 h after SAH.

### Determination of the effect of MT on H19, miR-675, let-7a, TP53, and NGF

**Fig. 7:**
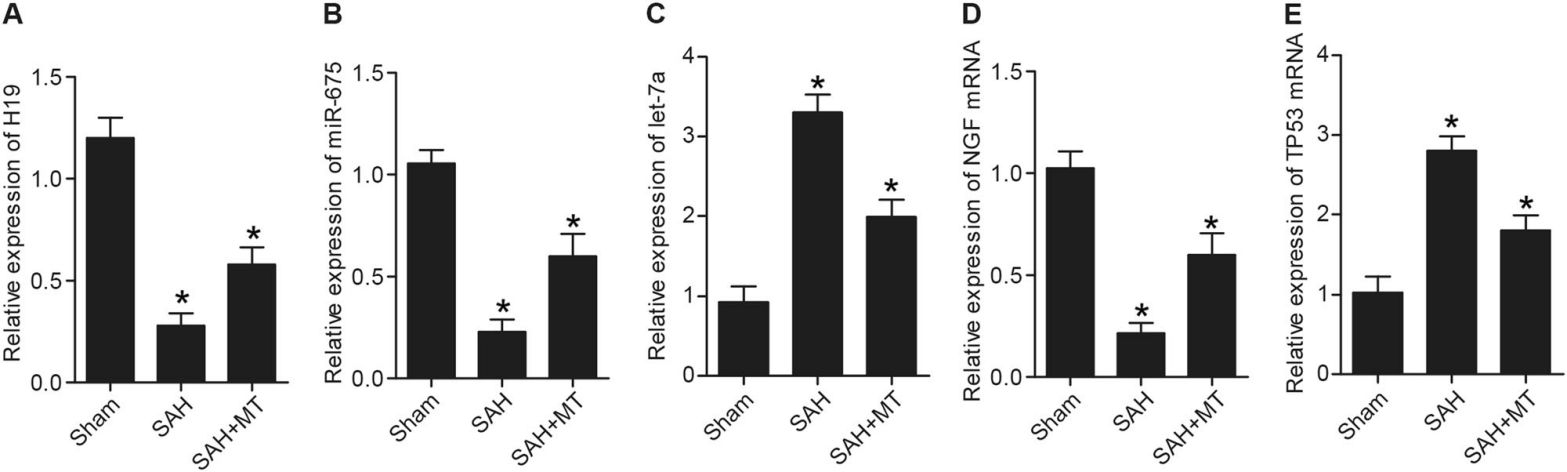
Effects of MT on H19, miR-675, let-7a, TP53, and NGF expressions were detected using real-time PCR and western blot analysis (*P < 0.05 compared with the sham group) **a** H19 level in the SAH group was much lower than in the SAH  +  MT treatment group; both of them were much lower than the sham group. **b** miR-675 level in the SAH  +  MT treatment group was much lower than control, and was even lower in the SAH group. **c** let-7a level in the SAH group was much higher than in the SAH  +  MT treatment group; both of them were much higher than the sham group. **d** NGF level in the SAH  +  MT treatment group was much lower than the sham group, and was even lower in the SAH group. **e** TP53 level in the SAH group was much higher than in the SAH  +  MT treatment group; both of them were much higher than the sham group

Real-time polymerase chain reaction(PCR) and western blot analysis were utilized to detect the effect of MT on H19, miR-675, let-7a, TP53, and NGF expressions. As shown in Fig. 7, levels of H19 mRNA (Fig. 7a), miR-675 mRNA (Fig. 7b), NGF mRNA, and protein (Fig. 7d) were much higher in the SAH  +  MT group than in the sham group, while the SAH group displayed even higher levels of H19 (Fig. 7a), miR-675 (Fig. 7b), and NGF (Fig. 7d). However, levels of let-7a mRNA (Fig. 7c), TP53 mRNA, and protein (Fig. 7e) were much lower in the SAH + MT group than in the sham group, while the SAH group displayed even lower levels of let-7a and TP53.

### Differential levels of TP53 and NGF among different groups

**Fig. 8:**
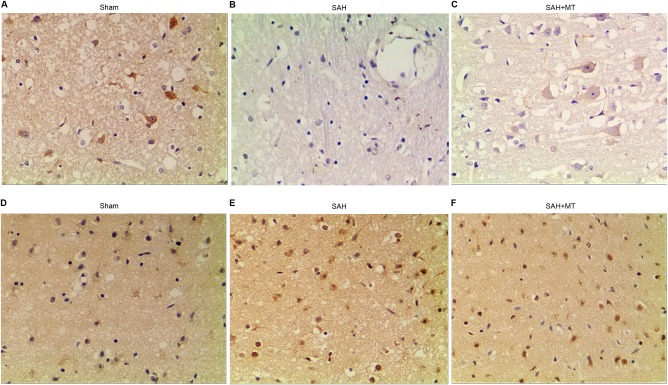
Levels of TP53 and NGF were different among different groups **a**−**c** NGF protein level in the SAH  +  MT treatment group was much lower than in the sham group, and was even lower in the SAH group. **d**−**f** TP53 protein level in the SAH group was much higher than in the SAH  +  MT treatment group; both of them were much higher than the sham group

Immunohistochemistry was conducted to measure protein levels of TP53 and NGF among the sham, SAH, and SAH  +  MT groups. As shown in Fig. 8, NGF protein level (Fig. 8a−c) in the SAH group was much lower than in the SHA + MT group, and both were much lower than the sham group; meanwhile, TP53 protein level (Fig. 8d−f) in the SAH group was much higher than in the SHA + MT group, and both of them were much higher than the sham group.

## Discussion

Melatonin has been reported to be used to treat neurological diseases, and the majority of this study has been focused on its ability to scavenger free radicals by promoting production of anti-oxidant enzymes and further initiating the synthesis of glutathione^23–26^. The focus of this present study was to explore its ability to attenuate signals of apoptosis, thereby preventing EBI following an event of SAH, by its interaction with non-coding RNA as well as their target genes. It has been shown that MT administration could alter the expression of H19, an lncRNA, that has been reported to be involved in the control of cellular apoptosis^27^. Recent studies have revealed that expression of H19 was substantially upregulated in response to a cerebral I/R injury^28^. In this study, we performed real-time polymerase chain reaction(PCR) and luciferase assay to investigate the effect of MT on transcriptional efficiency of H19 promoter, and we found that MT treatment upregulated H19 expression level, possibly by enhancing the transcriptional efficiency of H19 promoter.

Matouk et al. reported that the expression of Let-7a is up-regulated in the development of cerebral I/R brain^29^. If the upregulation was restored, the apoptosis as well as subsequent inflammation would be attenuated, lessening the injury to the nervous system^30^. Some studies have suggested that lincRNA is a precursor to other RNAs, which are notably smaller, with regulatory functionality. An example of a small RNA would be miRNAs. As it is understood that H19 is involved in apoptosis, it is not surprising that exon 1 is home to an miRNA. Closer examination of this miRNA indicates that there is a hairpin within it, providing a template for miR-675-5p and miR-675-3P^31^. In line with this, we confirmed that upregulation of H19 increased the expression of miR-675 in our study. Meanwhile, we also found that H19 is an endogenous competing RNA of let-7a by using luciferase reporter system. Furthermore, we searched the online miRNA database for the potential target of the H19-associated miRNAs (miR-675 and let-7a), and identified P53 or NGF as the virtual target gene of miR-675 or let-7a, respectively with the “seed sequence” in the 3′UTR, and the results of the following luciferase assay confirmed such regulatory association between the miRNAs and targets.

Accumulating evidence has indicated that SAH or other types of hemorrhage exposure inevitably induces cell death in human brains, and others have highlighted that inflammation occurring following the initial SAH could be a significant factor in the cell death that follows^32^. Our hypothesis was that MT treatment could ameliorate EBI by suppressing the apoptosis of neurons following an event of SAH by alternating the expression of the associated miRNAs such as miR-675 and let-7a.

Nerve growth factor is a neurotrophic factor and a neuropeptide that is primarily involved in the control of various functions including formation of synapses, and development, plasticity, growth, maintenance, apoptosis, and viability of neurons^33^. Neural growth factor has been shown to protect neurons against apoptosis by its ability to interact with some other anti-apoptotic genes such as PI3K, extracellular signal-related kinase/mitogen-activated protein kinase and phospholipase C pathways^33^. In addition, NGF has also been reported to protect the neurons against oxidative stress and toxin-induced apoptosis^33^. Another study indicated that NGF is capable of counteracting apoptosis, regardless of the causal factor^34^.

P53 is a known key tumor suppressor as it is a central hub where stress pathways are signaled. Therefore, it is effectively the region that controls the fate of human cells^35^. Apoptosis, or cell death, is valuable to maintain homeostasis when it is executed appropriately. For example, chemotherapeutic drugs and radiation are intended to place cellular stress on cancer cells and actively promote apoptosis^35^. Recent research indicated that p53 promoted the apoptosis via mitochondrial pathway, and this research is further supported by studies demonstrating that activation of Bax was mediated by suppressing p53^40^. Furthermore, activation of Bcl-2 protected against the apoptosis. It is also suggested that inhibiting Caspase 3 may be effective in preventing apoptosis, which would help to minimize the risk of EBI following an event of SAH^36^. In this study, we established the animal models of SAH, and found that MT treatment improved the neurological score and caused an evident decline in the brain swelling. Using TUNEL assay, we showed that MT inhibited apoptosis of neuronal in the brain cortex caused by SAH. Furthermore, the H19, miR-675, and NGF were decreased in response to SAH, while let-7a and TP53 were increased during this same process. Administration of MT to the SAH animal model partially, but significantly, restored the alternation of those ncRNAs and genes.

## Conclusion

Our study revealed for the first time that H19 regulates brain injury after SAH via microRNA -675 and let-7a by interacting with apoptosis induced by P53 and NGF. In this study, we found that administration of MT upregulated expression of H19, and H19 was shown to host miR-675, and miR-675 has been found to be a negative regulator of P53; meanwhile H19 was found to be a competing non-coding RNA for let-7a, and let-7a was found to be a virtual regulator of NGF. P53 and NGF were two important modulators of cell apoptosis, and finally influencing progression of brain injury after SAH. Our study revealed the involvement of H19-miR-675-P53-apoptosis and H19-let-7a-NGF-apoptosis signaling pathway in brain injury after SAH^38–40^.
